# Intrasellar pressure in patients with pituitary adenoma – relation to tumour size and growth pattern

**DOI:** 10.1186/s12883-022-02601-9

**Published:** 2022-03-09

**Authors:** Gabriel Simander, Per Olof Eriksson, Peter Lindvall, Lars-Owe D. Koskinen

**Affiliations:** 1 Department of Clinical Science-Neurosciences, Umeå University, 90187 Umeå, Sweden; 2 Department of Surgical Sciences Otorhinolaryngology, Uppsala University, Uppsala, Sweden

**Keywords:** Pituitary adenoma, Intrasellar pressure, Growth pattern, Classification

## Abstract

**Background:**

Only a few earlier publications on intrasellar pressure (ISP) have not been able to fully clarify any association between ISP and pituitary adenoma size and growth pattern. The aim of the study was to determine if intrasellar pressure (ISP) is elevated in patients with pituitary adenoma, and if the pressure is associated with tumour size and growth pattern.

**Methods:**

The study included 100 patients operated for suspected pituitary adenoma, who have had their ISP measured intraoperatively. All adenomas were classified on the basis of Knosp and SIPAP, from which further classification of invasiveness was performed. MRT examinations were used to calculate the tumour volume and diameter in three axes.

**Results:**

After exclusions, 93 cases were analysed. The mean ISP was 23.0 ± 8.4 mmHg. There were positive correlations between ISP and tumour volume and tumour diameters along all three axes. Coronal tumour diameter showed the strongest correlation with ISP elevation in a multivariate effect test. Adenomas classified as parasellar invasive (Knosp grade 3–4) showed higher mean ISP than adenomas considered as non-invasive (Knosp 0–2).

**Conclusions:**

ISP is affected by tumour anatomy and correlates positively with tumour volume. Tumour width, i.e. diameter in the coronal plane, appears to be the measure that most strongly affects the ISP. This is confirmed by the association between ISP elevation and parasellar growth.

## Background

Pituitary adenoma is one of the most prevalent of all tumours affecting the central nervous system. These tumours are benign, and often cause symptoms mainly associated with compression of surrounding structures. The role of intrasellar pressure (ISP) in patients with pituitary adenoma has been the subject of investigation in a few earlier publications, in which the ISP has been measured perioperatively to elucidate the role of ISP in relation to tumour size and symptoms of the patient. Although ISP has never been measured in healthy pituitaries, normal ISP is suggested to be similar to normal intracranial pressure (ICP). It is likely that a growing pituitary adenoma will cause an elevation of the ISP, and some publications have strengthened this hypothesis [1–3]. Earlier studies, which have explored the relation between ISP and adenoma growth pattern, have not been completely concordant, nor has an unambiguous correlation between ISP and tumour size been shown [4–6].

We assumed ISP to be elevated over suspected normal levels in patients with adenoma. We also hypothesized a correlation between ISP elevation and tumour size. At our clinic, ISP was measured during transphenoidal pituitary surgery between 2009–2015. The aim of the study was to evaluate ISP levels in patients with pituitary adenoma, and to determine if any association could be found between ISP and tumour size and/or growth pattern.

Preliminary results have previously been reported at the Scandinavian Society of Neurosurgery Congress 2013, Reykjavik, Iceland and at International Congress of Endocrinology 2018, Cape Town, Republic of South Africa.

## Methods

This study was a single-center, consecutive, retrospective, observational study with prospectively collected ISP data. Included in the study were patients with suspected pituitary adenoma who were operated at the Neurosurgical Department, Umeå University Hospital, Sweden between 2009–2015. All of these have had their ISP measured intraoperatively. This constituted a consecutively collected study population of 100 patients between 16–85 years old.

### Preoperative evaluation

All patients were preoperatively investigated according to the standardized procedure at our clinic. This included preoperative CT of the brain and sella, and MRI examination with and without gadolinium contrast enhancement. Endocrinologists and ophthalmologists evaluated each patient before and after surgery. Data were collected from patient records. On the basis of the preoperative radiological images, tumour characteristics including tumour diameter and growth pattern were acquired. Calculations of tumour measures in 3 axes (coronal, craniocaudal, anteroposterior) were performed. For tumour volume estimation, Automatic Sectra Volume Tool (Sectra Workstation, IDS7, ver 23.1) was used. All volume measurements were also manually checked.

Tumours were classified on the basis of Knosp [7] and SIPAP [8]-classifications. Tumour growth were thus graded in suprasellar, infrasellar, anterior, posteror (SIPAP) and parasellar (Knosp) direction. Tumours classified as parasellar grade 3–4 (Knosp) in one or both directions were considered as parasellar invasive, and therefore separated from tumours with parasellar grade 0–2 that were considered as not invasive [9].

### Intraoperative intrasellar pressure measurements

The surgical approach was transsphenoidal tumour extirpation, either microscope assisted through lateral rhinotomy, or by endoscopic assisted transnasal approach. All procedures during these years (2009–2015) were executed with one neuro-surgeon as the responsible main surgeon, and ISP measurements were performed as a part of the standardized surgical procedure at the clinic. Surgery was performed under standard neuro-anesthesia with oral intubation. Patients were normoventilated with pCO2-levels between 4.6–5.5 kPa. The ISP was measured before start of tumour resection using an intracranial pressure monitoring device (Codman® MicroSensor™ (CMS) Codman & Shurtleff Inc, Raynham, MA USA), which has been shown to accurately measure parenchymal pressure. This devices accuracy is well documented, and it is used clinically as a standard method for intracranial pressure monitoring [10–12]. A small bone window with a maximum of 2-mm diameter was performed in the sellar wall, followed by a 1–2 mm sharp cut of the dura to get access to the sellar room with extra attention to ensure no leakage of intrasellar content. After calibration the sensor was inserted into the tumour. ISP values were determined after pressure fluctuations had settled, and was noted in the surgical record. During pressure monitoring, patient´s head was held in plane position with the maximum elevation of 10°. The operation then proceeded with further removal of bone and tumour resection.

Histological diagnoses were defined according to the the routine histopatholocigal assessment at our hospital. Three adenomas had not a fully conclusive histochemical assessment and therefore classified as “histochemically unclear”, and was separated from the functioning and non-functioning adenoma group.

### Exclusions

Seven cases were excluded from the whole analysis, 6 of those because they had histological diagnoses other than pituitary adenoma. One subject was excluded because the anatomy of the sellar region had been completely destroyed by the pituitary adenoma, making it impossible to monitor the pressure accurately and draw any conclusion from the measurement. Thus, the final population consisted of 93 subjects. Of those, two cases were not included in tumour volume calculations, since their radiological images did not show enough quality for the software to performe acceptable and comparable volume estimations. A few cases (see Table 4) were inconclusive or had conflicting radiologic opinions regarding the anatomical classification, and were hence excluded from the separate classification analysis from which a distinct classification could not be done.

### Statistics

Patient characteristics including sex and age, tumour characteristics including tumour diameter in three axes and tumour volumes, are presented as means ± sd. ISP in the whole study population is presented as mean ± sd. Simple un-paired two-sided t-test was used to analyse any difference in ISP between groups of only two variables. ANOVA was used to analyse differences in ISP between groups of more than two variables. Pearson´s correlation was performed to evaluate the association between ISP and tumour diameter and tumour volume. Analysis of the three different tumour diameter axes´ effects on ISP, as well as multivariate analysis of preoperative characteristics in correspondence to ISP, were performed by multiple regression analysis. A p-value of < 0.05 was considered statistically significant. Statistical analyses were made using JMP Statistical Discovery ver. 14.2.

### Ethical considerations

The study was approved by the regional ethics review board. It was conducted in accordance with the World Medical Association Declaration of Helsinki entitled ‘‘Ethical Principles for Medical Research Involving Human Subjects”.

## Results

### Histological findings

Among the 100 patients included in the study, 94 had histopathological findings of pituitary adenoma. Other diagnoses were Rathke´s cleft cyst, unspecified cystic lesion, inflammatory lesion (2 cases), malignant sarcoma, and pituitary metastasis. After exclusions, the total study population constisted of 93 patients, all with pituitary adenoma diagnosis. The distribution of biochemical diagnoses are listed in Table 1. The majority (72%) of the adenomas were non-functioning.

**Table 1:** Histochemical distribution of adenomas

		Total	Men	Women	
		N	N	N	
Functioning adenoma					
	GH	12	7	5	
	Prolactin	2	1	1	
	ACTH	2	1	1	
	Thyroxine	1	1	0	
	Gonadotrophin	3	1	2	
	Multiple	3	1	2	
Non-functioning adenoma		67	39	28	
Functionality not clear		3	1	2	

Table 1 shows the histochemical distribution of the included pituitary adenomas. No difference in histochemical distribution, nor comparing functioning and non-functioning adenomas as groups, were found between men and women (ChiSquare-test)

The population consisted of 52 men and 41 women. The mean age was 61 ± 14 years (Table 2). Twenty-three were operated with microscope assisted approach and 70 endoscopically. Adenoma measures and volumes are presented in Table 3. The mean tumour volume was 6.13 ± 5.24 cm^3^. The mean coronal tumour diameter was 18.9 ± 5.1 mm, anteroposterior diameter 17.2 ± 5.7 mm, and craniocaudal diameter 21.9 ± 7.6 mm. The distribution based on the classifications is presented in Table 4*.* Thirty-six tumours showed parasellar growth in either or both directions classified as Knosp grade 3 or 4, and therefore defined as parasellar invasive. No differences in tumour volume, tumour measures or invasiveness were seen between men and women (Table 3). More men had tumours classified as P1 in the SIPAP-system compared to women, but no differences in classification in the other dimensions were seen between men and women. Thirteen adenomas showed radiological signs of pituitary apoplexy, i.e. hemorrhagic infarction. Of those, 9 cases had the typical clinical manifestation of apoplexy, including acute symptom onset or deterioration.

**Table 2:** Patient age and ISP

		Age	Intrasellar pressure (ISP)		
	N	Mean ± sd	Mean ± sd	95% CI mean	Min—Max
Total	93	61 ± 14 yrs	23.0 ± 8.4	21.3–24.7	11–54
Men	52	62 ± 14 yrs	24.6 ± 8.9	22.1–27.1	12–54
Women	41	59 ± 15 yrs	21.0 ± 7.3	18.7–23.3	11–40
*t-test*		*p* = *0.36*	*p* = *0.0368*		

Table 2 shows the mean age and mean intrasellar pressure (ISP) in the whole study population (total) and in men and women, respectively

**Table 3:** Pituitary adenoma volume and measures

Adenoma volumes and measures					
		Adenoma volume	Adenoma measures		
		(cm^3^)	Coronal (mm)	Anteroposterior (mm)	Craniocaudal (mm)
Total	Mean ± sd	6.13 ± 5.24	18.9 ± 5.1	17.2 ± 5.7	21.9 ± 7.6
	95% CI	5.03–7.22	19.0–20.0	16.0–18.4	20.3–23.5
	Min–Max	0.68–30.40	7.5–36.0	7.5–39.0	7.0–43.0
Men	Mean ± sd	6.39 ± 5.81	19.0 ± 5.6	18.0 ± 6.5	22.0 ± 7.4
	95% CI	4.76–8.02	17.4–20.6	16.3–19.9	20.0–24.1
	Min–Max	0.85–30.40	7.5–36.0	7.5–39.0	7.0–43.0
Women	Mean ± sd	5.80 ± 4.47	18.9 ± 4.4	16.1 ± 4.3	21.7 ± 8.0
	95% CI	4.37–7.23	17.5–20.3	14.7–17.4	19.2–24.3
	Min–Max	0.68–20.30	10.0–33.0	9.0–28.0	7.0–42.0
	*t-test*	*p* = *0.59*	*p* = *0.91*	*p* = *0.08*	*p* = *0.84*

Table 3 shows the mean adenoma volume and mean adenoma measures in three different axes. No difference in adenoma volumes or adenoma measure in any direction was found between men and women

**Table 4:** Tumours classified according to Knosp and SIPAP

**Classification**	**0**	**1**	**2**	**3**	**4**	*ChiSquare*
Suprasellar						
Total, *n* = 93	6	11	12	47	17	
Men, *n* = 52	3	6	10	27	6	*p* = *0.15*
Women, *n* = 41	3	5	2	20	11	
Infrasellar						
Total, *n* = 91	52	35	4			
Men, *n* = 51	27	21	3			*p* = *0.56*
Women, *n* = 40	25	14	1			
Parasellar dx						
Total, *n* = 91	16	32	21	16	6	
Men, *n* = 51	7	19	13	10	2	*p* = *0.57*
Women, *n* = 40	9	13	8	6	4	
Parasellar sin						
Total, *n* = 91	15	28	24	20	4	
Men, *n* = 51	6	15	15	11	4	*p* = *0.27*
Women, *n* = 40	9	13	9	9	0	
Anterior						
Total, *n* = 91	82	9				
Men, *n* = 51	44	7				*p* = *0.17*
Women, *n* = 40	38	2				
Posterior						
Total, *n* = 90	76	14				
Men, *n* = 50	38	12				*p* = *0.0135*
Women, *n* = 40	38	2				
Parasellar invasive	**Invasive**	**Non-invasive**				
Total, *n* = 91	36	55				
Men	21	30				*p* = *0.72*
Women	15	25				

Table 4 shows the adenoma classification according to Knosp and SIPAP. Adenomas with parasellar grade 3–4 were considered as parasellar invasive. ChiSquare-test was performed to analyse difference in the distribution between men and women

### Intrasellar pressure

**Fig. 1:**
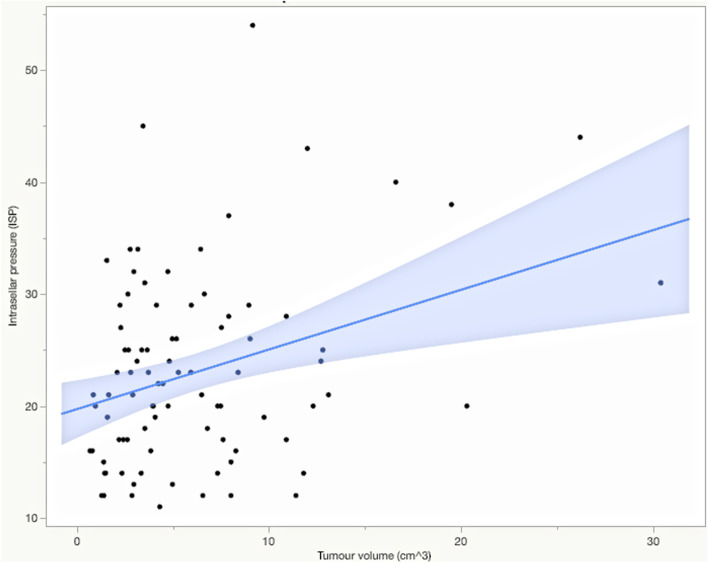
Intrasellar pressure in correlation to adenoma volume**.** *Pearson’s correlation. ISP showed a positive correlation to tumour volume, R* = *0.33 (p* = *0.0014)*

**Fig. 2:**
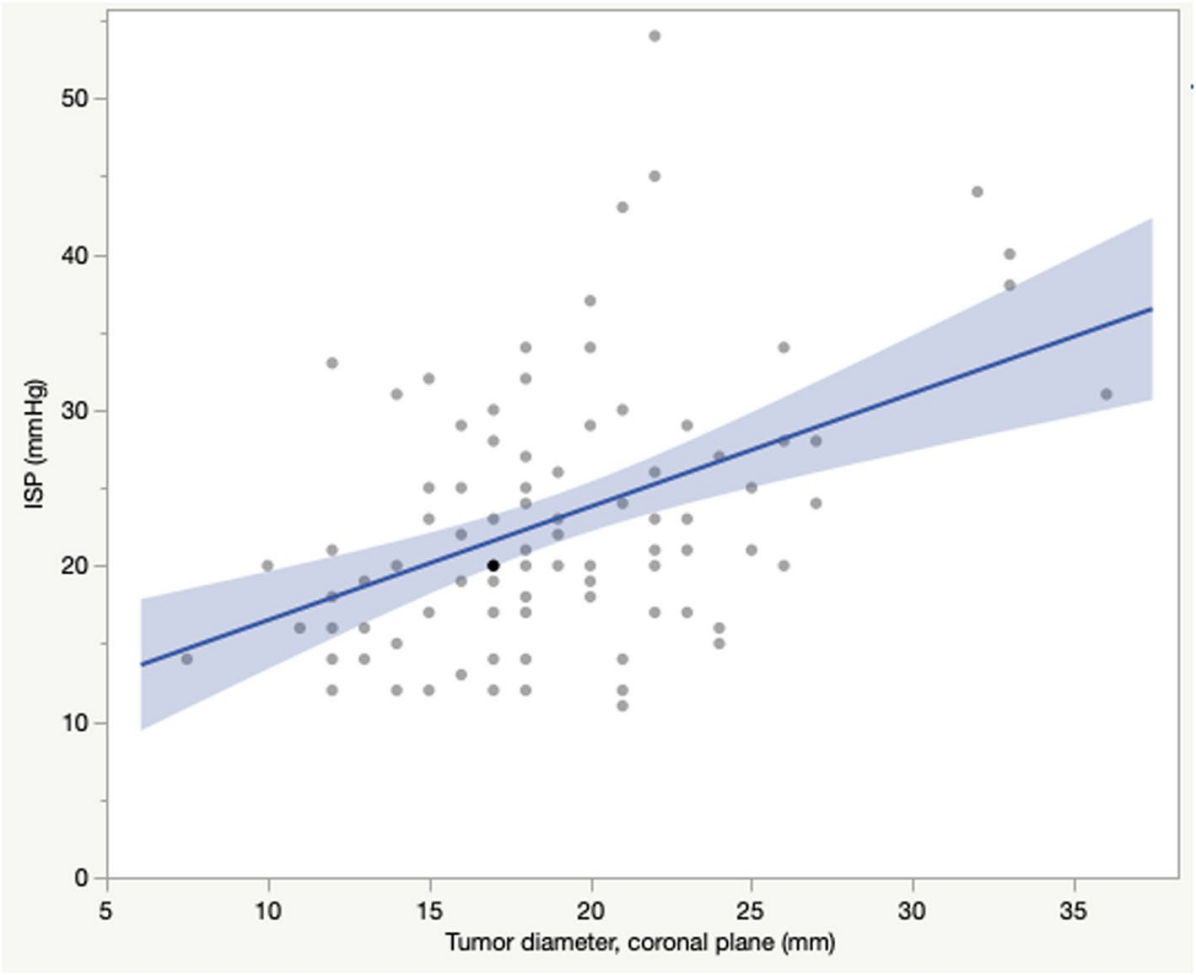
Intrasellar pressure in correlation to adenoma width. *Pearson’s correlation. ISP showed a positive correlation to coronal adenoma diameter (adenoma width), R* = *0.45 (p* > *0.0001)*

**Fig. 3:**
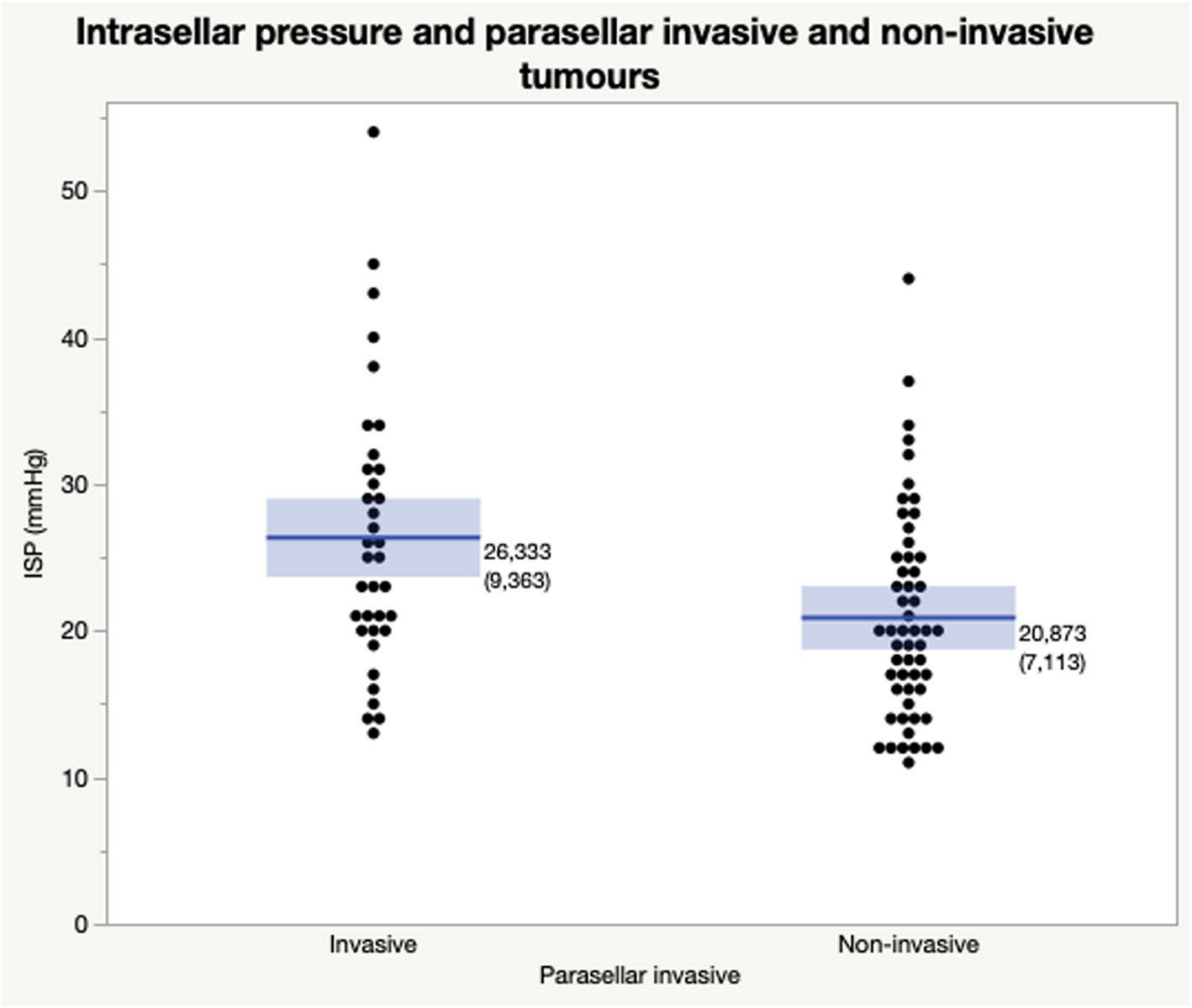
Mean intrasellar pressure (ISP) in adenomas growing invasively in parasellar direction compared to non-invasive adenomas. *Simple un-paired two-sided t-test. Adenomas with Knosp grade 3–4 (invasive) showed higher mean ISP (26.3 ± 9.4 mmHg) compared to adenomas with Knosp grade 0–2 (non-invasive), (21.9 ± 7.1 mmHg). (p* = *0.0022)*

The median ISP in the whole population (*n* = 93) was 21 mmHg, with a mean of 23.0 ± 8.4 mmHg. Men had higher mean ISP than women (24.6 ± 8.9 vs 21.0 ± 7.3 mmHg, *p* = 0.0368) (Table 2)*.* There was a positive correlation between ISP and tumour volume (R = 0.33, *p* = 0.0014) (Fig. 1). Furthermore, correlation between ISP and tumour diameter was seen in all of the three compared axes: the coronal tumour diameter (R = 0.45, *p* < 0.0001*)* (Fig. 2), the anteroposterior tumour diameter (R = 0.32, *p* = 0.0021), and the craniocaudal tumour diameter (R = 0.23, *p* = 0.0277). In the multivariate effect test including all diameters the coronal tumour diameter showed the strongest effect in the model (*p* = 0.0007). Tumours classified as parasellar invasive (Knosp grade 3–4, *n* = 36) had higher mean ISP (26.3 ± 9.4 mmHg) than tumours classified as Knosp grade 0–2 (21.9 ± 7.1 mmHg, *n* = 55) (*p* = 0.0022) (Fig. 3).

There were no significant difference in ISP when comparing the other growth directions of the SIPAP-system, i.e. suprasellar-, infrasellar-, anterior- and posterior growth, respectively.

ISP showed no correlation to age. Neither were there any difference in ISP between the different surgical approaches. No difference in mean ISP was found in patients with acute apoplexy (22.9 ± 10.3 mmHg, *n* = 9) compared to patients without (23.0 ± 8.2, *n *= 84) (*p* > 0.05).

The results from univariate analyses were confirmed by an additional multivariate regression analysis exploring ISP in correlation to the basic characteristics (age, sex, tumour volume, tumour invasiveness and apoplexy). In this model sex (*p* = 0.0350), tumour volume (*p* = 0.0140), and parasellar invasiveness (*p* = 0.0249) still showed significant effect on ISP.

Notable is also that two of the excluded subjects, one with pituitary sarcoma and one with granulomatous inflammation had very high ISP, 51 mmHg and 42 mmHg, respectively.

## Discussion

From this series we have a clear indication that ISP is elevated in patients with pituitary adenoma. We have demonstrated that ISP can be correlated to or predicted by the tumour anatomy, and that ISP correlates positively to tumour volume. Tumour width, i.e. diameter in the coronal plane, appears to be the measure that most strongly reflects ISP. This is also confirmed by the association between ISP elevation and grade of parasellar growth defined by the Knosp classification.

Although ISP in completely healthy pituitaries is not known, there is reason to believe that pituitary adenoma can lead to elevation of ISP above suspected normal levels. Lee et al. 1994 contributed to the hypothesis of the normal ISP being similar to the known normal intracranial pressure (ICP), by performing ISP measurements on patients with empty sella and patients with bromocriptine treated microadenoma, acquiring ISP levels below 15 mmHg [5]. The results of our study correspond well to ISP levels in sellar tumours presented in earlier studies, ranging between 17 and 33 mmHg [3–5, 13], and support the hypothesis that ISP in these patients is elevated above known regular intracranial pressure [14] and hypothesized normal intrasellar pressure. To put the acquired ISP levels into any contaxt, it could be of relevance to mention that studies on ICP have shown levels above 20 mmHg to be potentially dangerous to the brain tissue [15]. Interesting is also the fact that the mean ISP exceeds the normal central venous pressure [16], and that important supply to the pituitary is mediated by a venous portal system with theroretically limited resistance to external pressure [17].

The relationship between tumour volume and ISP was investigated by Gondim et al. 2006, who found the highest ISP levels in large tumours which still had not grown through the sellar wall [4]. In a newly published study (2018) by Hayashi et al, greater ISP levels were found in small tumours (diameter < 26 mm) than in large tumours (diameter > 26 mm), but no difference was found when comparing ISP in relation to cavernous sinus invasion [18]. Some earlier publications indicate an association between raised ISP and parasellar tumour growth pattern [2, 5]. The series performed by Arafah et al. in 2000, though, did not show any association between ISP and tumour volume [6]. In this context of rather unconcordant previous reports on this subject, our results illustrate a direct correlation between tumour volume and ISP elevation, which has not been shown in any of the previous studies in this field. Furthermore, we have been able to show that ISP corresponds positively with tumour width, i.e. coronal diamater, which has been demonstrated as the measure of greastest impact on ISP compared to the anteroposteror and craniocaudal diameter.

To further discuss how tumour growth pattern affects ISP, we found higher ISP levels in tumours with an invasive parasellar growth into the cavernous sinus, in this study defined as Knosp grade 3–4. This result is in line with the conclusion of the tumour width being the measure that most strongly influences the ISP. Knosp’s classification is today the widely used system for classification of parasellar growth. However, definition of invasiveness from MRI is not unproblematic since there are reports showing that invasiveness can not be excluded in tumours graded as Knosp < 3 [9, 19, 20] It has, though, been described high certainity of invasiveness to the cavernous sinus among tumours graded Knosp 3–4, why this definition was used in this study. Our results strengthens the hypothesis that an invasive growth pattern is associated to high ISP, rather than tumour growth without invading through the sellar walls, which has been the alternative assumption previously presented.

One possible explanation for these findings, firstly postulated by Lee et al.[5], is that the parasellar growth pattern brings the tumour in closer contact with the carotid artery, which might enable direct pressure transmission, as well as development of arterial neovascularization from smaller branches. This theory could explain why there has not been proven equal ISP elevation from tumours with direct infrasellar invasion, where the intrasellar pressure theoretically is equilibrated with the atmospheric pressure in the sphenoid sinus. One may also speculate that rigid bone structures have to be expanded with tumour growth into the parasellar region, and to do so the ISP has to be elevated. Another factor of interest is how ISP is affected by the tumour´s growth rapidity. It is rational to assume that a fast-growing tumour gives rise to more aggressive pressure elevation, and also may be associated to a higher grade of invasive potential of the tumour. It has been described high ISP levels measured in patients operated after acute deterioration due to pituitary apoplexy, in which the pathophysiology is based on ischemic necrosis and haemorrhage. This is believed to be caused by, and further develop, rapidly increased pressure within the sella [1]. In this study we could not detect higher mean ISP in subjects who had pituitary apoplexy. At our institution, patients with severe symptoms from acute apoplexy were generally operated within 24 h, which certainly raises the question whether higher ISP levels would be seen if measured closer in time to symptom onset or deterioration. Noteworthy is that, in the previous report by Zayour et al, patients with high ISP after pituitary apoplexy were operated as late as 1 week after symptom onset. However, a somewhat larger subgroup of cases with acute pituitary apoplexy in our series would have strengthen the conclusive potential of our results in this matter.

A finding of interest is that this study demonstrates higher mean ISP in men compared to women. Similar findings are not reported in previous publications where ISP have been explored. There have, though, been reported gender differences in some parts of the clinical course of pituitary adenoma disease [21], for example that women with prolactinoma has been shown to seek medical advice earlier and with smaller adenomas than men, which probably is a result of the fact that menstrual cycle interruptions often present as an initial symptom. On the other hand, in cases with other adenoma types, some reports state that men have tumours that are smaller and less invasive at time of surgery, and also an advantage in outcome [22]. Others have shown adenoma invasiveness to be associated with male gender [9, 23]. For this reason, analyses comparing adenoma characteristics have been performed in this study population without obtaining any differences between men and women concerning factors which we know have the potential to affect ISP, for example tumour size or invasiveness. Thus, an obvious explanation behind this result cannot be concluded, and indicates the need of further investigations.

This study presents one of the largest study populations presented so far in this field. ISP measurements have been performed in a standardized manner at one centre, by one responsible neuro-surgeon. The method chosen for the ISP measurements is similar to that used in the majority of previous studies, and the accuracy of the device used is satisfactorily documented. Is there a risk of pressure decrease following durotomy? We do not think so because the actual dural opening in our study was initially only big enough for the insertion of the monitoring device. Special attention was taken toward ensuring that no leakage of intrasellar content followed the insertion. As in every case concerning this type of invasive pressure monitoring, it is not possible to exclude a small pressure decrease, but as long as studies are performed according to similar methodology, results are still comparable.

Our results further explore the significance of tumour anatomy in terms of ISP, and may serve as a ground for understanding the pathophysiological mechanisms of pituitary tumourous disease. Further clinically relevant questions that are raised with these results is how ISP corresponds to preoperative symptoms and postoperative outcome. Sella constitutes a confined space with crucial structures in the close surrounding, including cranial nerves and the low-pressure venous portal system that regulates the important functions of the pituitary. One may speculate that increased ISP leads to a microcirculatory deterioration affecting the pituitary function and surrounding tissues, that in turn may be associated to hormonal imbalances, endocrine recovery potential and postoperative complications.

## Conclusions

ISP seems to elevated above suspected normal levels in patients with pituitary adenoma. ISP is positively correlated with tumour volume and tumour width. We could not confirm an earlier study that found the highest ISP levels in adenomas confined in the sella. Coronal tumour diameter seems to have a greater impact on ISP than growth in the other directions.

## Authors’contribution

LOK contributed to the study conceptualization and design. LOK, POE and PL contributed to data collection. Data analysis was performed by GS and LOK. The manuscript was written by GS and LOK. All authors have read, commented on, and approved the final manuscript.

## Data Availability

Original data are held by the authors and are available on request.

## Abbreviations

ISP: Intrasellar pressure
ICP: Intracranial pressure
